# Age-stratified outcomes of laparoscopic hiatal hernia repair with Nissen fundoplication in children: a single-center experience

**DOI:** 10.1007/s00383-026-06480-w

**Published:** 2026-06-13

**Authors:** Kutay Bahadir, Ege Evin, Ilayda Sagpazar, Ege Ekiyor, Ergun Ergun, Gulnur Gollu, Meltem Kologlu, Murat Cakmak, Ufuk Ates

**Affiliations:** 1 Department of Pediatric Surgery, Ankara University Faculty of Medicine, Ankara, Turkey; 2 Department of Pediatric Surgery, Kırıkkale Yüksek Ihtisas Hospital, Kırıkkale, Turkey

**Keywords:** Hiatal hernia, Laparoscopy, Nissen fundoplication, Pediatrics, Recurrence

## Abstract

**Purpose:**

To evaluate outcomes of laparoscopic hiatal hernia repair with Nissen fundoplication in children, comparing infants (≤ 12 months) and older children (> 12 months).

**Methods:**

Pediatric patients who underwent laparoscopic hiatal hernia repair with Nissen fundoplication between 2008 and 2025 were retrospectively reviewed. Patients were classified by age at surgery into ≤ 12 months and > 12 months. Outcomes included postoperative complications, recurrence, length of hospital stay, and follow-up.

**Results:**

Nineteen patients were included in this study (10 boys, 9 girls); of which 9 were ≤ 12 months and the remaining 10 were > 12 months. Age-stratified postoperative outcome comparisons were performed in 17 patients after exclusion of 2 patients (%10.5) with concomitant gastrostomy. Median age at repair was 20 months (IQR 5–58.5; 5 days–120 months) and median weight was 9.8 kg (IQR 5.1–17.5; 2.4–50.0 kg). Median length of stay was 4 days (IQR 3–5; 2–7 days), and median follow-up was 84 months (IQR 38.5–112; 4–205 months). Selective mesh reinforcement was used in 3 patients (15.8%) with wide hiatal defects and/or poor crural tissue quality. Postoperative complications occurred in 4 patients (21.1%). Recurrence occurred in 3 patients (15.8%) at 2, 4, and 9 months postoperatively. All underwent redo laparoscopic repair, and no further recurrence was observed during follow-up.

**Conclusion:**

Laparoscopic hiatal hernia repair with Nissen fundoplication appeared feasible in selected pediatric patients, including infants ≤ 12 months. In this small single-center cohort, recurrences occurred within the first postoperative year and were managed with redo laparoscopy. These findings should be interpreted cautiously and require confirmation in larger multicenter studies.

## Introduction

Hiatal hernia is a medical condition characterized by the abnormal protrusion of the upper part of the stomach and/or other internal organs through a defect in the diaphragmatic hiatus, often presenting with nonspecific findings ranging from feeding intolerance and vomiting to respiratory symptoms and recurrent pulmonary infections [1, 2]. Although hiatal hernia is often diagnosed due to clinical suspicion following pathological presentation, incidental detection on imaging is not uncommon. On chest radiography, visualization of a retro-cardiac air–fluid level is usually suggestive of gastric fundus herniation into the posterior mediastinum. Physical examination alone is not sufficient for diagnosis as it is frequently non-specific. Initial diagnostic tests for hiatal hernia include chest radiography and an upper gastrointestinal contrast study [3]. Indications for surgical repair include persistent symptoms, development of complication, imaging-confirmed clinically significant hernia, however pediatric evidence remains limited, and practice patterns vary across centers [1].

Over the last decade, minimally invasive repair has been increasingly favored in the field of pediatric surgery, but recurrence, especially those requiring reintervention remain major concerns. So far, the literature across pediatric series suggests that recurrence may occur despite an apparently intact wrap, likely as a result of progressive widening or failure of the hiatal closure [1]. In parallel, the outcomes of pediatric fundoplication operations are heterogeneous, and long-term follow-up studies continue to show clinically significant rates of recurrence and redo procedures following antireflux surgery [4, 5]. Of the various forms of available fundoplication techniques, current evidence suggests that Nissen fundoplication is favored [6, 7].

Age at repair is frequently discussed as a potential modifier of outcome. Baseline physiology, comorbidity burden and hiatal tissue characteristics of infants may differ from those of older children, yet data focusing on early-life repair using consistent operative technique is rare. Reporting of recurrence is also not uniform across studies, with some being symptom-driven, while others rely on imaging, further complicating comparisons [8].

We reviewed cases of laparoscopic hiatal hernia repair combined with Nissen fundoplication in our center and compared intraoperative as well as follow-up outcomes between infants (≤ 12 months) and older children (> 12 months). The primary outcome of interest was recurrence; the secondary included postoperative complications and length of hospital stay.

## Materials and methods

This single-center retrospective cohort study included a total of 19 pediatric patients who underwent laparoscopic hiatal hernia repair with anti-reflux surgery between 2008 and 2025 at a tertiary referral center. Over the study period, 24 children underwent operative repair for hiatal hernia at our institution. Five patients treated with a primary open (laparotomy) approach were excluded, and the remaining 19 patients who underwent laparoscopic repair with Nissen fundoplication comprised the analytic cohort. The study was conducted in accordance with the Declaration of Helsinki and was approved by the Institutional Ethics Committee of Ankara University School of Medicine (Approval No: I11-1060-25; Date: January 5, 2026).

**Fig. 1:**
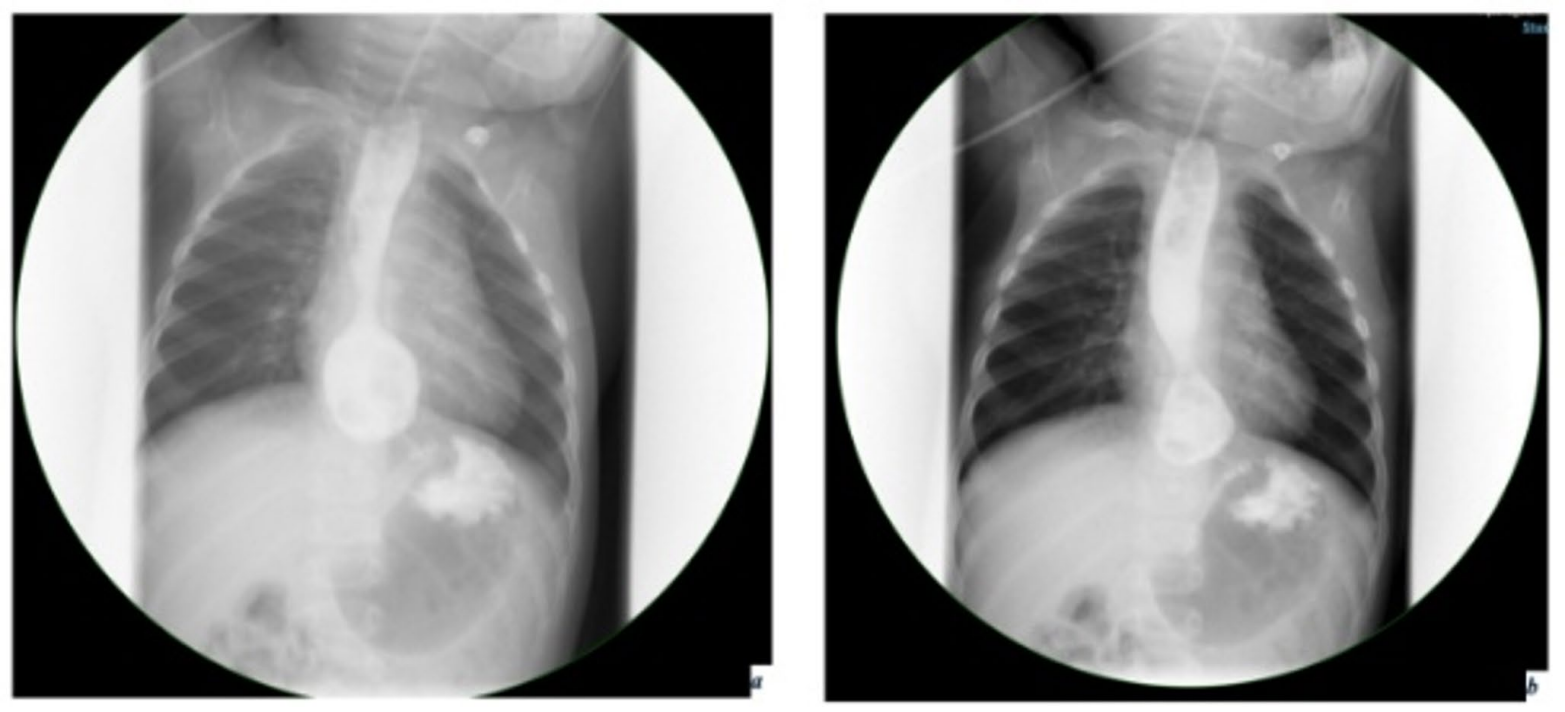
Preoperative contrast esophagograms obtained at two different time points in a patient with a type I (sliding) hiatal hernia. Both images demonstrate upward displacement of the gastroesophageal junction above the diaphragm, consistent with a sliding hiatal hernia, with intrathoracic herniation of the proximal stomach

**Fig. 2:**
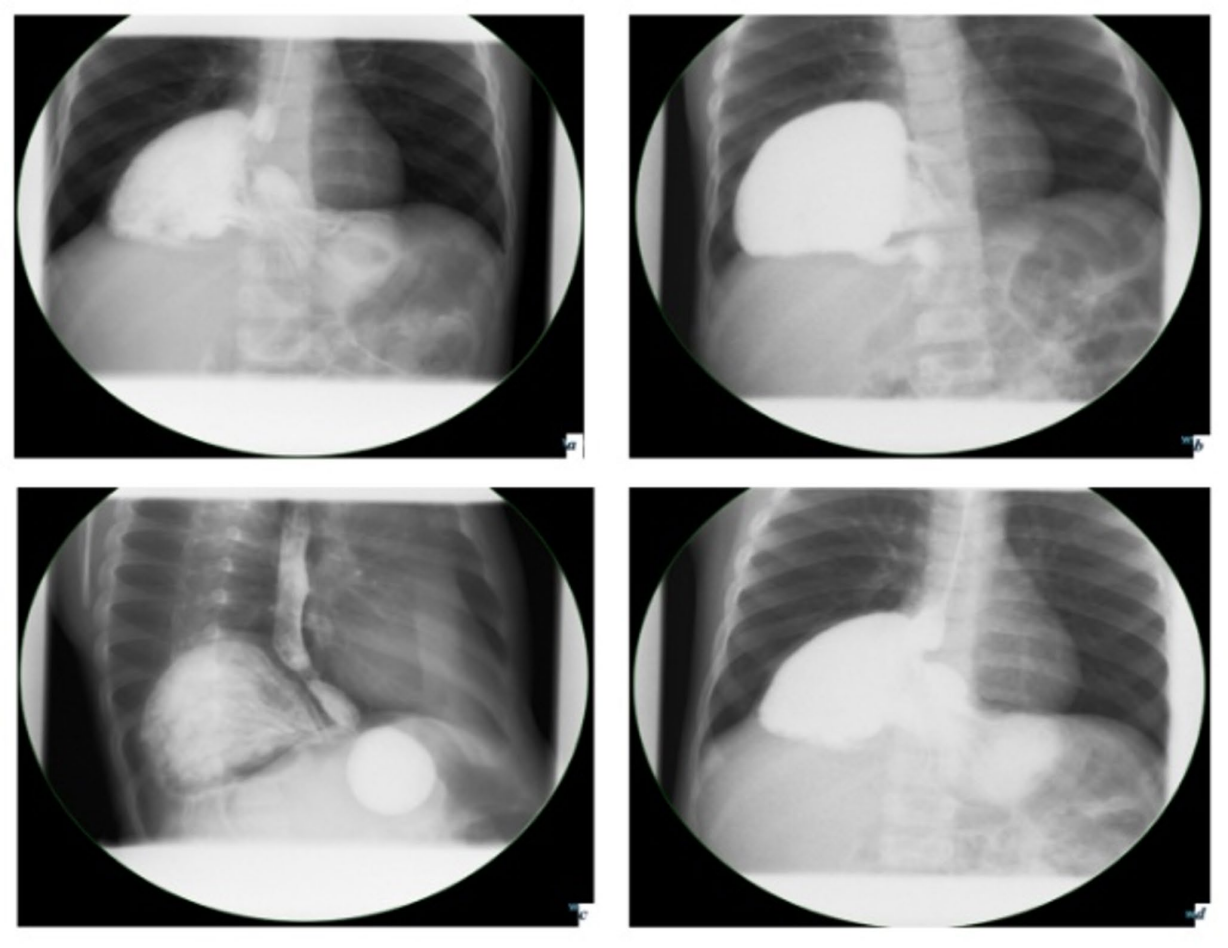
Preoperative contrast esophagograms obtained in different projections and at different time points in a patient with a type III (mixed) hiatal hernia. **a**, **b** Anteroposterior views demonstrating intrathoracic herniation of the gastric fundus with a large contrast-filled gastric pouch above the diaphragm. **c** Lateral view showing upward displacement of the gastroesophageal junction together with herniation of the proximal stomach into the thoracic cavity. **d** Repeat anteroposterior view confirming the persistent intrathoracic position of both the gastroesophageal junction and the gastric fundus

**Fig. 3:**
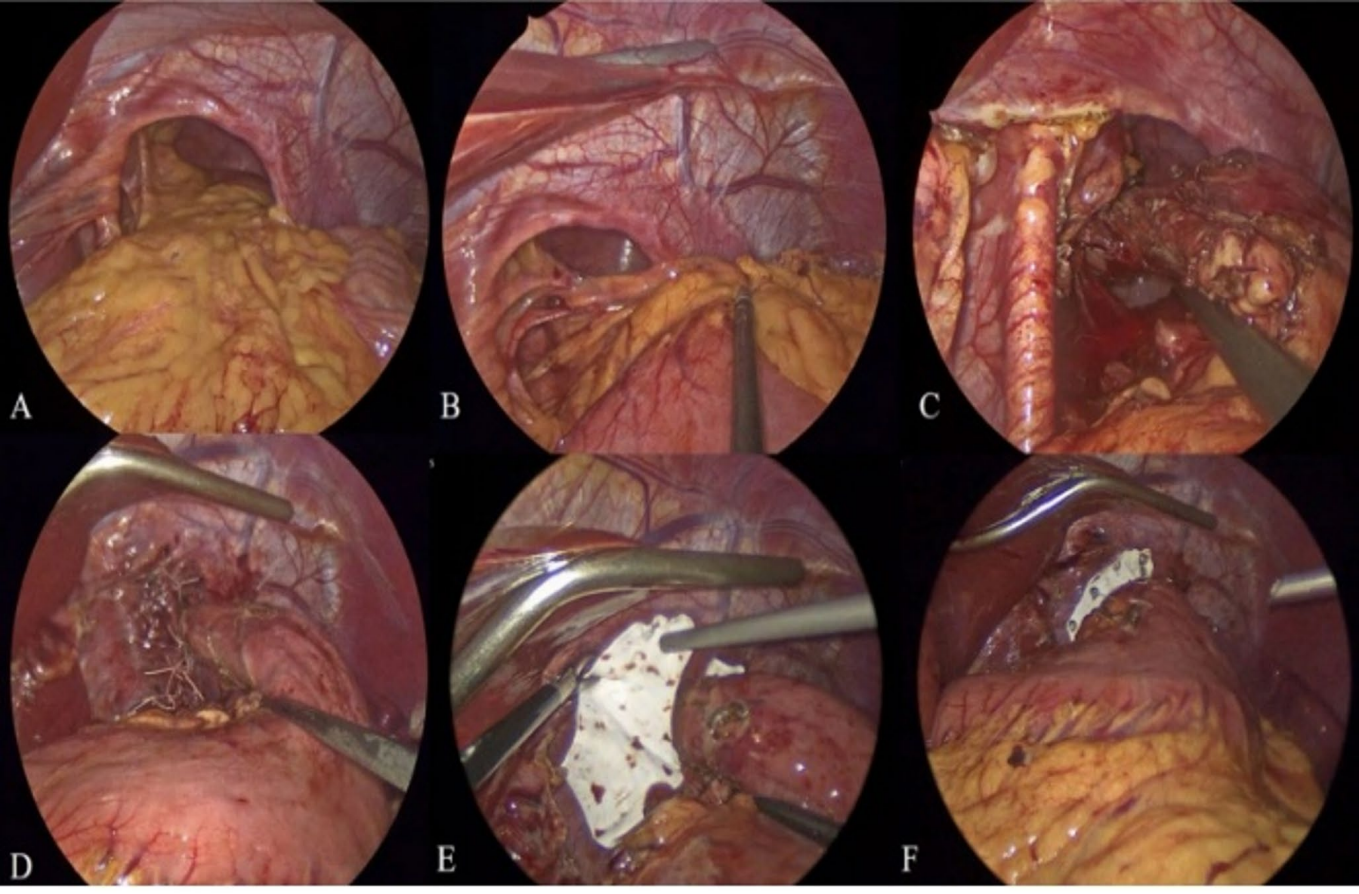
Surgical steps of laparoscopic hiatal hernia repair and Nissen fundoplication. **A** Exposure of the hiatus after liver retraction using a Nathanson retractor, demonstrating the herniated gastric and omental tissue. **B** Reduction of the herniated stomach and omentum from the thoracic cavity into the abdominal cavity. **C** Dissection of the peritoneum overlying the esophagus and mobilization of approximately a 2-cm segment of the abdominal esophagus. **D** Following dissection of the peritoneum over the diaphragmatic crura, hiatal closure (cruroplasty) was performed using six 2/0 Ethibond sutures, taking care not to constrict the esophagus. **E** Placement and fixation of a mesh over the narrowed hiatal area using a laparoscopic tacker. **F** After creating a window behind the abdominal esophagus, completion of a 360° Nissen fundoplication by wrapping the fundus around the esophagus. The fundoplication was constructed with three 2/0 Ethibond sutures: the first as stomach–stomach, the second as stomach–stomach–esophagus–diaphragm–stomach, and the third as stomach–esophagus–stomach

Patients were divided into two subgroups: Group 1 (Age ≤ 12 months) and Group 2 (Age > 12 months). Inclusion criteria were: (1) patients under the age of 18 years, and (2) a definitive diagnosis of hiatal hernia based on upper gastrointestinal contrast study and/or computed tomography (CT) (Figs. 1 and 2) and (3) underwent laparoscopic hiatal hernia repair with fundoplication concomitantly. Exclusion criteria were as follows: (1) age older than 18 years, (2) lack of preoperative informed consent from guardians, (3) incomplete clinical data and (4) a primary open (laparotomy) approach.

Demographic data, hernia subtype, surgical approach, intraoperative findings, postoperative complications (including recurrence, gastroesophageal reflux, postoperative dysphagia), and follow-up duration were obtained from the institutional medical records. Recurrence was defined as reappearance of a hiatal hernia confirmed on follow-up imaging (upper gastrointestinal contrast study and/or CT), including the routine postoperative third-week contrast study, or additional imaging obtained when recurrence was suspected clinically. Postoperative dysphagia and GER symptoms were positively counted only when clearly documented in the inpatient charts or outpatient follow-up notes.

### Preoperative protocol

Patients with suspected hiatal hernia underwent diagnostic confirmation with an upper gastrointestinal contrast study and/or CT, as clinically indicated (Figs. 1 and 2). Preoperative upper gastrointestinal endoscopy was not performed routinely. Endoscopy was reserved for selected patients with suspected mucosal disease, gastrointestinal bleeding, severe or refractory reflux symptoms, or dysphagia; therefore, endoscopic findings were not included as a standardized preoperative variable. All patients received a standardized preoperative assessment including a complete physical examination and routine laboratory testing (complete blood count, serum electrolytes, liver and renal function tests, and coagulation profile). Bowel preparation was performed preoperatively with a rectal enema at a dose of 20 mL/kg. Single-dose intravenous cefazolin was administered prophylactically within 60 min prior to skin incision.

### Surgical techniques

All procedures were performed under general anesthesia with endotracheal intubation, and patients were positioned supine throughout the operation. An esophageal bougie was not utilized. After induction of anesthesia, an age-appropriate nasogastric tube was placed for decompression and maintained throughout the operation. Laparoscopic repair was performed using a multi-port technique. A 4-mm or 5-mm camera trocar was inserted into the peritoneal cavity via the Hasson technique through an umbilical incision. Insufflation pressure was maintained between 4 and 12 mmHg; lower pressures were preferred in neonates and small infants to minimize ventilatory and hemodynamic compromise. Two 3-mm or 5-mm working ports were placed under direct vision. A Nathanson liver retractor was used to elevate and retract the left hepatic lobe, thereby improving upper abdominal exposure and facilitating safe dissection of the hiatus and gastroesophageal junction. The herniated sac was dissected and reduced into the abdomen. Circumferential mobilization of the distal esophagus was achieved to provide adequate intraabdominal length. The diaphragmatic crural edges were approximated posteriorly with interrupted non-absorbable sutures (Ethibond Excel^®^ Polyester; Ethicon, Inc.). After adequate esophageal mobilization, the gastric fundus was wrapped posteriorly around the abdominal esophagus through a newly created retroesophageal window and fashioned into a 360° Nissen wrap, which was then secured to the diaphragm using non-absorbable sutures (Fig. 3). The abdominal layers were closed in anatomical planes.

### Postoperative protocol and follow-up

Postoperative management was standardized for both age groups. The nasogastric tube was removed on the day of surgery. Oral intake was initiated with clear liquids on postoperative day 1, followed by gradual advancement of diet as tolerated. Analgesia was provided using paracetamol at a dose of 10–15 mg/kg per administration. Patients were discharged from the hospital when they were in an afebrile, pain-free state and were able to tolerate oral feeding. An upper gastrointestinal contrast study was performed at postoperative week 3 to assess for recurrence.

The patients were followed further in our outpatient clinic. Follow-up information was obtained retrospectively from routinely documented outpatient clinic visits and institutional electronic medical records as part of standard clinical care. After discharge, patients were followed up in the outpatient clinic on the 3rd week, 3rd month, 6th month and 1st year of surgery.

### Statistical analysis

Statistical analyses were performed using IBM SPSS Statistics for Windows (IBM Corp., Armonk, NY, USA). Patients were classified according to age at surgery into those ≤ 12 months and those > 12 months of age. Because concomitant gastrostomy may influence postoperative feeding, length of hospital stay, and early recovery, the two patients who underwent gastrostomy were retained in the descriptive cohort but excluded from age-stratified comparative postoperative outcome analyses. Continuous variables were assessed for distribution and are reported as median (interquartile range [IQR]) and range; between-group comparisons were performed using the Mann–Whitney U test. Categorical variables are presented as number (percentage) and were compared using Fisher’s exact test. Univariable associations with recurrence were summarized using odds ratios (ORs) with 95% confidence intervals (CIs); when zero cell counts were present, a continuity correction was applied. All tests were two-sided, and p values < 0.05 were considered statistically significant.

## Results

Over the study period, 24 children underwent operative repair for hiatal hernia at our institution; 5 patients treated with a primary open (laparotomy) approach were excluded, leaving 19 laparoscopic cases with Nissen fundoplication for analysis. A total of 19 patients underwent a total of 22 operations, reflecting the need for reoperation due to recurrent hernia in a subset of patients. Patients were classified by age at surgery into ≤ 12 months (*n* = 9) and > 12 months (*n* = 10). Of these, 52.6% (*n* = 10) were male and 47.4% (*n* = 9) were female. The median age at repair was 20 months (IQR 5.0–58.5; range 5 days–120 months) and the median weight was 10.4 kg (IQR 5.6–19.9; range 2.4–50 kg).

Presenting symptoms were not mutually exclusive and included vomiting, cough and respiratory distress (Table 1). Preoperative diagnosis was supported by an upper gastrointestinal contrast study in 14 (73.7%) and by CT in 10 (52.6%). Type 3 hernia accounted for 10 (52.6%) of cases (type 1: 7 (36.8%), type 2:1 (5.3%) and type 4 (with omentum): 1 (5.3%)). Among patients with classifiable hernia type (type 3 vs. type 1), recurrence occurred in 3/10 of the type 3 cases and in 0/7 type 1 cases (*p* = 0.228; odds ratio 7.00, 95% CI 0.31–160.32) (Table 2).

**Table 1:** Baseline demographic and clinical characteristics of patients according to age at surgery

Variable	≤ 12 months (*n* = 9)	> 12 months (*n* = 10)	*p* value
Age at surgery, months	4.0 (IQR 1.0–7.0)	58.5 (IQR 47.5–79.8)	< 0.001
Weight at surgery, kg	4.7 (IQR 3.2–6.9)	19.9 (IQR 12.2–23.8)	< 0.001
Preoperative contrast study positive	7/9 (77.8%)	7/10 (70.0%)	1.000
Preoperative CT positive	6/9 (66.7%)	4/10 (40.0%)	0.370
Concomitant gastrostomy	1/9 (11.1%)	1/10 (10.0%)	1.000
Symptoms			
Vomiting	4/9 (44.4%)	6/10 (60.0%)	0.656
Cough	1/9 (11.1%)	5/10 (50.0%)	0.141
Respiratory distress	0/9 (0.0%)	5/10 (50.0%)	0.033*
Incidental detection	4/9 (44.4%)	2/10 (20.0%)	0.350

Data are presented as median (interquartile range, IQR) for continuous variables and as number (percentage) for categorical variables. Categorical variables were compared using Fisher’s exact test; continuous variables using the Mann–Whitney U test. *Bold values indicate statistical significance (p<0.05)

**Table 2:** Univariable analysis of factors associated with recurrence

Factor	Recurrence / Total	Odds ratio	95% CI	*p* value
Age ≤ 12 months vs. > 12 months^*^	1/8 vs. 2/9	0.50	0.04–6.68	1.000
Preoperative type 3 vs. type 1^*^	3/12 vs. 0/5	4.05	0.17–93.99	0.515
Mesh reinforcement at primary repair (yes vs. no^†^**)**	0/3 vs. 3/16	0.55	0.02–13.36	1.000

Data are presented as recurrence/total number of patients in each group. Odds ratios (ORs) and 95% confidence intervals (CIs) were calculated using Fisher’s exact test, and a continuity correction was applied when zero cell counts were present

*Calculated after exclusion of patients with concomitant gastrostomy

†Descriptive subgroup analysis in the overall laparoscopic cohort

Crural repair and fundoplication were performed in all patients and concomitant gastrostomy was performed in 2 patients (10.5%). Because these procedures could affect postoperative feeding and recovery parameters, these two patients were excluded from the age-stratified comparative outcome analysis. In three patients (15.8%), mesh reinforcement (Gore-Tex^®^ DualMesh^®^, W. L. Gore & Associates, USA) was selectively applied to the crural repair due to the presence of a wide hiatal defect and/or poor crural tissue quality on intraoperative assessment (Fig. 3). No recurrence was observed among patients who underwent primary mesh reinforcement (0/3), whereas recurrence occurred in 3 of 16 patients without primary mesh reinforcement; given the small number of mesh cases, this comparison was considered descriptive. Oral intake was initiated at 24 h postoperatively according to the institutional postoperative protocol. Median length of stay was 4 days (IQR 3–5; range 2–7), and median follow-up was 84 months (IQR 38.5–112; range 4–205). After exclusion of the two patients who underwent concomitant gastrostomy, age-stratified outcome analysis included 17 patients (≤ 12 months, *n* = 8; >12 months, *n* = 9). Recurrence rates remained similar between infants and older children (1/8 [12.5%] vs. 2/9 [22.2%], *p* = 1.000), and no significant differences were observed in postoperative complications, GER symptoms, dysphagia, length of stay, or follow-up duration (Table 3). All patients had a normal routine postoperative third week upper gastrointestinal contrast study and subsequently presented with clinical symptoms, with the diagnosis of recurrence confirmed by repeat contrast imaging.

**Table 3:** Comparison of postoperative outcomes according to age at surgery

Outcome	≤ 12 months (*n* = 8)	> 12 months (*n* = 9)	*p* value
Recurrence	1/8 (12.5%)	2/9 (22.2%)	1.000
GER symptoms	1/8 (12.5%)	2/9 (22.2%)	1.000
Dysphagia symptoms	1/8 (12.5%)	2/9 (22.2%)	1.000
Length of stay, days	4 (IQR 3–5)	4 (IQR 3–5)	0.921
Follow-up duration, months	106.0 (IQR 29.8–133.2)	84.0 (IQR 39.0–111.0)	0.810

Patients who underwent concomitant gastrostomy were excluded from this comparative outcome analysis. Categorical variables were compared using Fisher’s exact test; continuous variables were compared using the Mann–Whitney U test

All recurrences were attributed to reherniation through the hiatus, with an intact fundoplication wrap in all cases, and all patients underwent redo laparoscopic repair without any subsequent recurrence. Among the three patients with recurrence, mesh reinforcement was used during the redo repair in only one patient. No recurrence was observed in patients who had undergone mesh-reinforced crural repair during the primary operation.

## Discussion

In this single-center cohort of 19 children who underwent laparoscopic hiatal hernia repair with Nissen fundoplication, recurrence was observed in 15.8% of patients and was detected within the first postoperative year. All recurrent cases underwent redo laparoscopic repair, and no further recurrence was documented during the available follow-up. Given the limited cohort size, these findings should be interpreted as descriptive rather than definitive. Nevertheless, the pattern of recurrence in our series supports the view that recurrent hiatal hernia may often reflect failure at the level of the hiatal closure rather than disruption of the fundoplication wrap.

The reported recurrence rates following pediatric hiatal hernia repair vary widely in the literature, in part because the observed cohorts differ in hernia size and/or type, associated comorbidities, if any, as well as follow-up strategy. Recent pediatric data continues to demonstrate that recurrence remains a clinically relevant point of interest even in contemporary minimally invasive practice. Engall and colleagues noted that reported recurrence rates in pediatric series have varied widely and they reported a 13% recurrence rate in a recent case series examining the outcomes of hiatoplasty and fundoplication performed in patients diagnosed with large hiatal hernias [9]. The overall recurrence rate in our cohort is similar to those frequently reported in contemporary pediatric literature, albeit direct comparisons remain a challenge due to differences in recurrence definitions and surveillance strategies. Gang et al. highlighted heterogeneity in diagnostic modality and follow-up in their pediatric experience, and noted that recurrence, be it radiologic or symptomatic may not uniformly prompt reoperation [1]. Similarly as underscored by the SAGES 2024 guideline, the variability in evidence regarding key operative adjuncts – such as mesh reinforcement- and the necessity of individualized management reflects that even in well-studied adult settings, recurrence remains a common complication and yet, its definition continues to vary across centers and publications [10].

Recurrence appeared to cluster in anatomically more complex hernias. In our cohort, all recurrences occurred in type III hernias (3/10), whereas no recurrence was observed among type I hernias (0/7). Although this difference did not reach statistical significance and should not be overinterpreted, the observed distribution is clinically plausible. Larger or mixed hiatal hernias may place greater mechanical demand on crural closure, particularly when tissue quality is poor or the repair is performed under tension. This observation is consistent with the concept that hiatal anatomy, rather than the fundoplication alone, may be an important determinant of anatomic durability. Recent pediatric and foregut series similarly suggest that recurrence is often related to hiatal failure or reherniation rather than isolated wrap disruption [1, 9, 11, 12].

The comparative outcomes of total versus partial fundoplication remains a topic of widespread controversy in pediatric surgery. Karpelowsky et al. reported that a substantial proportion of children who did not undergo a concomitant antireflux procedure at the time of hiatal hernia repair subsequently developed clinically significant gastroesophageal reflux requiring further surgical intervention [13]. Hu et al. retrospectively and comparatively analyzed a cohort of 136 children who underwent laparoscopic hiatal hernia repair with Nissen-Rossetti or versus those who underwent repair with concomitant Thal fundoplication over a 13-year period [14]. The data highlights the broad comparability of rates of long-term failure between laparoscopic complete (Nissen) versus partial (Thal) fundoplication, with no statistically significant difference in the ‘absolute’ failure in a randomized cohort followed beyond 10 years [15]. Trade-offs specific to the fundoplication technique have nevertheless been reported; in some series, a higher burden of dysphagia/stricture has been reported after Nissen–Rossetti, whereas Thal fundoplication has been linked to higher rates of postoperative reflux recurrence [14, 16]. Total (Nissen) fundoplication remains widely favored in antireflux surgery and is now predominantly performed laparoscopically [17]. Despite providing good reflux control, function-limiting sequela such as gas-bloat symptoms and dysphagia is consistently reported in a subset of patients [18]. In our series, a standardized ‘floppy’ Nissen fundoplication was performed in all patients. The wrap was fashioned with minimal tension with the aim of reducing the probability of postoperative dysphagia and gas-bloat symptoms development. Notably, postoperative dysphagia and GER-related symptoms were observed only in the first two patients of the series, whereas no such complications occurred in the subsequent 17 patients. This temporal pattern suggests a learning-curve effect, reflecting the transition from a relatively tighter wrap toward the “floppy” Nissen technique, which is relatively tension free. Our experience supports the concept that paying meticulous attention to construction of the wrap and avoiding excessive tension are two critical determinants of functional outcomes following total fundoplication.

Mesh reinforcement has been used in hiatal and paraesophageal hernia repair to provide additional support to the crural closure, particularly in large defects or recurrent cases; however, the evidence remains heterogeneous, and current guidelines do not support routine mesh use for all patients [10–12]. In children, the threshold for mesh placement is even higher because of concerns regarding growth, foreign-body reaction, erosion, and uncertain long-term safety; therefore, available pediatric experience supports a selective rather than routine approach [1, 9]. In our series, mesh reinforcement was used selectively in three patients during the primary operation because of a wide hiatal defect and/or poor crural tissue quality. No recurrence was observed in these patients; however, the number of cases is too small to draw conclusions regarding the protective effect of mesh. In all recurrent cases, reherniation occurred through the hiatus while the fundoplication wrap remained intact, suggesting that the dominant mechanism of recurrence was related to the hiatal repair rather than wrap disruption. These findings support careful intraoperative assessment of the crura but do not establish an indication for routine mesh use in children. Larger pediatric series are needed to clarify the long-term safety and potential role of selective reinforcement.

Age at repair is often considered a potential modifier of outcome, particularly in infants, who may differ from older children in comorbidity burden, respiratory status, and tissue characteristics [1, 19]. In our cohort, recurrence, postoperative complications, and length of hospital stay did not differ significantly between infants (≤ 12 months) and older children (> 12 months). However, the small number of patients in each subgroup limits the strength of this comparison. Therefore, these findings should not be interpreted as evidence that age has no effect on outcome, but rather as an observation that laparoscopic repair was feasible in both age groups within our institutional experience. The higher frequency of respiratory distress among older children may reflect age-related differences in symptom expression and referral patterns rather than a distinct anatomical risk profile. Taken together, our findings are consistent with contemporary pediatric experience suggesting that laparoscopic hiatal hernia repair may be feasible in selected children, including infants [1, 19].

The main limitation of this study is the small number of patients. This limits the statistical power of subgroup comparisons and precludes reliable multivariable adjustment for potential confounders such as comorbidity burden, hernia type, objective hiatal defect size, number of crural sutures required, degree of intrathoracic stomach, crural tissue quality, intraabdominal esophageal length after mobilization, and perceived tension at crural closure. For this reason, the associations observed in this series, particularly those related to age group, type III hernia, and selective mesh reinforcement, should be considered descriptive and hypothesis-generating rather than confirmatory. Additional limitations include the retrospective single-center design, incomplete uniform recording of operative time, and the lack of standardized long-term imaging beyond the early postoperative period. Nevertheless, the study reflects a consistent minimally invasive approach with standardized fundoplication and predefined early postoperative imaging. Because preoperative upper gastrointestinal endoscopy was not part of the routine diagnostic pathway during the study period, objective endoscopic assessment of esophagitis or mucosal pathology was not uniformly available.

In conclusion, laparoscopic hiatal hernia repair with Nissen fundoplication appeared feasible in selected pediatric patients, including infants. Recurrence occurred in 15.8% of patients, presented within the first postoperative year, and was managed with redo laparoscopy. The clustering of recurrence in type III hernias suggests that hiatal anatomy may be relevant to anatomic durability; however, the limited sample size prevents definitive conclusions. Larger multicenter studies with standardized recurrence definitions and follow-up protocols are needed.

## Data Availability

No datasets were generated or analysed during the current study.
